# Organic matter degradation and bacterial communities in surface sediment influenced by *Procambarus clarkia*

**DOI:** 10.3389/fmicb.2022.985555

**Published:** 2022-10-21

**Authors:** Yiran Hou, Rui Jia, Peng Ji, Bing Li, Jian Zhu

**Affiliations:** ^1^Key Laboratory of Integrated Rice-Fish Farming Ecology, Ministry of Agriculture and Rural Affairs, Freshwater Fisheries Research Center, Chinese Academy of Fishery Sciences, Wuxi, China; ^2^Wuxi Fisheries College, Nanjing Agricultural University, Wuxi, China

**Keywords:** *Procambarus clarkii*, organic matter degradation, bioturbation, fatty acid, sediment, aquaculture pond

## Abstract

To alleviate excessive organic matter (OM) accumulation in sediments and reduce the risk of endogenous water pollution and eutrophication in aquaculture ponds, an 84-day experiment investigated the effect of the red swamp crayfish *Procambarus clarkii* on the OM degradation and bacterial communities in sediments. The experiment established two groups, *P. clarkia* treatment and control (represented as PG and CG, respectively), with three replicates for each group. At the end of experiment, the total, light fraction, and heavy fraction organic matter concentrations in the sediment of the PG group were significantly lower than those of the CG group. Significantly higher oxidation–reduction potential (ORP) and more extensively degraded OM, indicated by fatty acids, were observed in the PG group. Compared to the CG group, the average OM removal efficiency induced by crayfish in the PG group was 15.24%. Using 16S ribosomal RNA (rRNA) high-throughput sequencing, we investigated the differences in benthic bacterial communities between the PG and CG groups. Linear discriminant analysis (LDA) effect size (LEfSe) analysis revealed that Nitrospirae, Nitrospira, Alphaproteobacteria, OLB14, Nitrospirales, Rhodobacterales, Rhizobiales, Micrococcales, Nitrospiraceae, Rhodobacteraceae, *Nitrospira, Rhodobacter, Thermomonas*, and *Denitratisoma* were significantly enriched in the PG group. Four significantly different functional groups related to OM degradation were determined between the PG and CG groups according to the functional annotation of procaryotic taxa (FAPROTAX) analysis. These four functional groups, aerobic chemoheterotrophy, manganese oxidation, dark iron oxidation, and dark sulfide oxidation, showed significantly higher relative abundances in the PG group. Overall, *P. clarkia* effectively increased the ORP values of sediments to provide favorable conditions for OM degradation and changed the composition and function of bacterial communities to improve bacterial abilities for OM decomposition, thereby promoting OM degradation in the sediment.

## Introduction

Aquaculture plays an important role in food security and meets the increasing global demand for nutritious foods (Costello et al., 2020). Aquaculture production worldwide reached 82.10 million tons in 2018, and aquaculture ecosystems, including farming ponds, lakes, reservoirs, and rivers, have become increasingly important (Food and Agriculture Organization of the United Nations, 2020). However, large amounts of artificial nutrient input in aquaculture ecosystems always lead to environmental stress and other issues, especially in farming ponds. High stocking density and feed input result in low resource utilization efficiency and considerable residual organic waste in farming ponds (Cao and Wang, 2010; Liu, 2011; Liu et al., 2011; Deng and Jiang, 2013). The residual organic waste consists mainly of uneaten food, feces, and other organic detritus, part of which is discharged along with tail-water, thereby inducing nutritional pollution and even eutrophication in the surrounding aquatic ecosystems (Ji et al., 2000; Zhou et al., 2011). Most of the residual organic waste accumulate on the sediments of farming ponds through settlement, complexation, and adsorption (Cao and Wang, 2010; Liu, 2011; Deng and Jiang, 2013).

The accumulation of organic waste in sediment carries the risk of endogenous water pollution, which is also a potential threat to pond ecosystems and the surrounding aquatic environment (Diaz, 2001; Sanz-Lázaro and Marín, 2008). Although the sediment is an important carrier, storage, and buffer area for the migration and transformation of biogenic elements, it is gradually transformed into a release source of nutrients and toxic substances such as hydrogen sulfide as the organic matter (OM) continuously deposits and decomposes anaerobically in farming ponds, thus causing endogenous water pollution (Christensen et al., 2000; Gray et al., 2002; Hargrave et al., 2008; Carlsson et al., 2012). However, studies focusing on inhibiting OM accumulation and sediment remediation in farming pond ecosystems are still limited compared with the increasing number of studies on water purification and tail-water treatment in farming ponds (Lin et al., 2002; Van Rijn, 2013; Flora and Kröger, 2014; Li et al., 2017; Li X. et al., 2018; Wu et al., 2018; Shao et al., 2020). Meanwhile, the methods used in actual production to remove the accumulated OM in sediments have certain deficiencies, such as high mechanical operating costs, pollution, or harm to water bodies and aquatic animals, long phytoremediation cycles, and limitations due to natural environmental conditions (Wang et al., 2013).

Based on the feeding habits and ability to utilize organic debris of some economically valuable macrobenthos, researchers have tried to reduce sedimentary OM accumulation in aquaculture pond by co-culturing the sea cucumber *Apostichopus japonicus*, freshwater prawn *Macrobrachium nipponense*, or snail *Bellamya purificata* (Yuan et al., 2008; Ren, 2012; Ge, 2014; Hou et al., 2021b). Benthic fauna plays an important role in aquatic ecosystems, especially in the biogeochemical cycle at the sediment–water interface, as they can affect physical structures and chemical properties of the sediment and accelerate material exchange and transformation at the sediment–water interface (Aller, 1988; Pelegrí and Blackburn, 1994; Murray et al., 2002; Stockdale et al., 2009; Zhang et al., 2010; Sanz-Lázaro and Marín, 2011). Additionally, benthic fauna can affect the composition, diversity, metabolism, and function of benthic bacterial communities (Mermillodblondin et al., 2004; Laverock et al., 2010; Rugenski et al., 2012; Hou et al., 2021a). Changes in the bacterial community and environmental factors affect OM degradation in sediments. Hence, comprehensive exploration of benthic fauna, OM degradation, environmental conditions, and microbial communities will help to better understand the bioremediation potential of benthic organisms to inhibit organic pollutants. However, related studies are still limited, and the pathways and mechanisms of specific species affecting the OM degradation process still need to be studied.

Red swamp crayfish *Procambarus clarkia* is an endemic North American crayfish species introduced worldwide and is the most popularly cultivated freshwater crayfish (Gutiérrez-Yurrita and Montes, 2001; Cheng, 2019). As a typical benthic organism and opportunistic omnivorous feeder, *P. clarkia* is considered to be a generalist and a keystone species in aquatic ecosystems, which causes transformations in the ecological function and environmental structure of aquatic habitats owing to its endemic trophic behavior and feeding habits (Ilhéu and Bernardo, 1993; Lodge and Hill, 1994; Momot, 1995; Gutiérrez-Yurrita et al., 1998, 2017; Angeler et al., 2001; Gutiérrez-Yurrita and Montes, 2001; Correia, 2002). *Procambarus clarkia* can rapidly deplete all food sources available owing to its voracity and ability to shift its diet, resulting in dramatic effects on the entire aquatic ecosystem, including altering benthic food webs, sediment quality, habitat structure, and benthic algae (McCormick, 1990; Ilhéu and Bernardo, 1993; Lodge et al., 1994; NystrÖM et al., 1996; Gutiérrez-Yurrita et al., 1998). This aggressive voracity and adaptability of *P. clarkia* may make co-culturing *P. clarkia* in aquaculture ponds an effective way to solve the excessive OM accumulation in pond ecosystems with high artificial nutrition input. However, there is little evidence on how crayfish affect OM degradation, nutrient cycles, sediment characteristics, and bacterial communities.

In this study, we used fatty acids (FAs) and Illumina high-throughput sequencing of 16S rRNA genes to evaluate OM degradation states and to estimate bacterial community structures in sediments, respectively (Vonk et al., 2008; Li F. et al., 2018). Our study aimed to investigate the effects of crayfish *P. clarkia* on sediment characteristics, sedimentary OM degradation, and benthic bacterial communities; determine the interactions and interrelationships between sediment characteristics, OM degradation, and bacterial communities; and explore the pathways and mechanisms of *P. clarkia* affecting OM degradation. In addition, we hope to provide theoretical support for inhibiting OM accumulation and preventing water pollution in aquaculture ponds and surrounding aquatic ecosystems.

## Materials and methods

### Culture experiment

The experiment was conducted at the Freshwater Fisheries Research Center of the Chinese Academy of Fishery Sciences (Wuxi, China). The experimental crayfish *Procambarus clarkii* and sediments were obtained from a breeding farm (Wuxi, China). The crayfishes were healthy with an average weight of 60.07 ± 0.80 g, and were kept in glass tanks for 14 days to become acclimatised to the laboratory conditions before the experiment. The experimental sediments were dried in the sun, ground to a powder in a mortar, passed through a 100-μm mesh sieve, and mixed evenly before use to maintain consistency and homogeneity (Nie et al., 2011; Wang et al., 2015; Hou et al., 2017). Six glass tanks (63 cm × 30 cm × 39 cm) were used in the experiment and divided into two groups: three replicates for the *P. clarkia* treatment and three for the control (represented as PG and CG, respectively). All the glass tanks were covered with a 5-cm thick layer of sediment at the bottom and then filled with freshwater. The experimental freshwater used was tap water that had been filtered and fully aerated. The sediment in each tank was precipitated and stabilized for 14 days before the experiment. After acclimation, nine crayfish were randomly and equally divided into three glass tanks designated for the PG, whereas no crayfish were allocated to the CG. For the other experimental conditions, the PG and CG were strictly consistent. The water temperature and dissolved oxygen were kept at 26.5 ± 0.5°C and 6.5 ± 0.2 mg/L during the experimental period, respectively. The experimental lighting condition was a natural light/dark cycle. Commercial feed (Zhejiang Haida Feed Co., Ltd., China) was used as experimental diets after being ground and sieved through a 100-μm mesh, which contained total organic matter (123.40 ± 1.15 mg/g), total nitrogen (43.2 ± 1.23 mg/g), and total phosphorus (7.18 ± 0.34 mg/g). At 4:00 p.m. every day, 5.4 g diets (equal to 3% of six crayfish body weight) were repeatedly weighed six times, mixed well with freshwater, and evenly poured into six tanks. The experiment lasted for 84 days without any water exchange. All crayfish maintained good vitalities without any mortality.

### Sample collection

Sediment samples were collected at the beginning and end of the experiments. We randomly selected nine sampling points in each glass tank and used a plastic pipe measuring 2 cm in diameter to collect 0–1 cm surface sediment samples. In addition, nine sediment samples from the same glass tank were thoroughly mixed. The sediment samples for evaluating the bacterial communities were immediately stored in a freezer at −80°C until analysis. The sediment samples for determining the total organic matter (TOM), light fraction organic matter (LFOM), heavy fraction organic matter (HFOM), and fatty acid (FA) were dried in a lyophilizer (CHRIST LYO Alpha 1-4 LD plus, Germany) at −50°C for 72 h, ground and homogenized in a mortar, and then stored in a freezer at −80°C until analysis.

### Sediment properties determination

According to the density, the TOM in the sediment can be divided into LFOM and HFOM. LFOM generally has a density of less than 2.0 g cm^−3^ and is mainly composed of newly formed and easily degradable animal, plant, and microbial residues (Haynes, 2000; Jiang et al., 2019). HFOM mainly includes refractory organic matter adsorbed on the mineral surface or masked in soil aggregates (Bao et al., 2013). In comparison, LFOM is susceptible to natural and artificial disturbances and is more sensitive and rapid in responding to environmental variations (Haynes, 2000; Huang et al., 2020). Therefore, t TOM content was estimated by loss on the ignition after being ashed at 400°C for 4 h (Martinez-Garcia et al., 2015). The LFOM was determined using a modified version of the method described by Janzen et al. (1992). Approximately, 5 g of dried sediment sample was weighed and placed into a 50 ml centrifuge tube with 20 ml NaI solution (specific gravity = 1.70 ± 0.02). After shaking for 1 h and centrifuging at 4,200 r·min^−1^ for 10 min, LFOM was collected from the suspension using Whatman no. 1 paper. NaI solution (20 ml) was added to the centrifuge tube, and the same procedure was repeated three times to isolate and collect LFOM. All the collected LFOM was then washed under suction with 200 ml of 0.01 mol·L^−1^ CaCl_2_ and distilled water. After being dried at 60°C for 24 h, the collected LFOM was scraped from Whatman filter paper and weighed. The HFOM was obtained by subtracting the LFOM from the TOM. The average OM removal efficiency induced by crayfish at the end of the experiment was calculated using the following formula:

Average OM removal efficiency = (TOM_CG-FNL_ – TOM_PG-FNL_)/TOM_CG-FNL_ × 100%

TOM_CG-FNL_ and TOM_PG-FNL_ indicate the average TOM contents in the sediments of the CG and PG groups at the end of the experiment, respectively. In addition, the oxidation–reduction potential (ORP) of the sediment was measured at 1-cm depth intervals at the beginning of the experiment and then after 2, 4, 8, and 12 weeks, using a portable multi-parameter analyzer (Hach HQ4300 Multi/ISE/3 Channels).

### Fatty acids extraction and quantification

Fatty acids were extracted from freeze-dried and homogenized sediment samples according to the methods described by Ding and Sun (2005) and Lopes et al. (2018). Approximately, 2 g sediment sample was weighed, extracted with 10 ml methanol in a 50 ml glass centrifuge tube, and then extracted with 10 ml methylene chloride–methanol (2:1, v/v) three times. During each extraction, the mixture was sonicated for 10 min and centrifuged at 2,500 rpm for 8 min at 4°C to separate the phases. All supernatants were combined and partitioned into the methylene chloride phase by washing with a 5% NaCl solution. The methylene chloride phase was collected and evaporated under a N_2_ atmosphere. Once the methylene chloride phase was dried, 10 ml 0.5 mol/L KOH/Methanol was added to saponify the lipids at 80°C for 2 h to release the neutral and acidic lipids. Neutral lipids were extracted from the basic solution (pH > 13), and FAs were extracted after the addition of HCl (pH < 2). The FAs in all extracts were esterified with 2 ml 25% BF3-Methanol to form fatty acid methyl esters (FAMEs). Before gas chromatography analysis, an internal standard (undecanoic acid methyl ester for FAMEs) was added to the extracts.

The FAMEs were analyzed using a gas chromatograph (GC-2030, Shimadzu) equipped with an on-column injector (GC-2030, Shimadzu), flame ionization detector (GC-2030, Shimadzu), and capillary column (DB-WAX, Agilent; 30 m × 0.32 mm i.d.). The oven temperature was programmed to start at 100°C for 3 min, then increased to 180°C at 10°C/min, maintained at 180°C for 1 min, increased to 240°C at 3°C/min, and finally maintained at 240°C for 9 min. N_2_ was used as the carrier gas at a flow rate of 3 ml/min. The temperatures of the injection and flame ionization detectors were both 250°C. The injection volume was 0.6 μl, and the split ratio was 1:10. Each FAME was identified by comparing the retention times with those of 37 fatty acid methyl ester mixed standards (Supelco, United States). The concentration of each FA in the sediment (mg fatty acid per dry weight sediment) was quantified using undecanoic acid methyl ester as an internal standard.

Based on the concentration of each fatty acid in the sediment, the ratio of long-chain (C > 20) vs. short-chain (C < 20) alkanoic acids (RLS) and the ratio of unsaturated C18:1–3 n-alkanoic acids (including C18:1, C18:2, and C18:3) to C18:0 n-alkanoic acid (C18:1-3/C18:0) were calculated to evaluate the OM degradation state in the sediment (Li F. et al., 2018).

### 16S rRNA gene amplification and sequencing

Total DNA was extracted from the sediment samples according to the manufacturer’s protocols using the E.Z.N.A.® Soil DNA Kit (Omega Bio-Tek, Norcross, GA, United States). The DNA quality and quantity were determined using a NanoDrop 2000 Spectrophotometer (Bio-Rad Laboratories Inc., United States). The V3-V4 regions of the 16S rRNA genes from each extracted DNA sample were amplified using primers 341F-806R (341F: ACTCCTACGGGAGGCAGCAG; 806R: GGACTACHVGGGTWTCTAAT; Berg et al., 2012). All PCRs were performed in 15 μl reaction volumes containing 7.5 μl of Phusion ®High-Fidelity PCR Master Mix (New England Biolabs, Ipswich, MA, United States), 1 μl of forward and reverse primers (10 μM), 1 μl of dNTPs (2.5 mM), and 1 μl template DNA. Thermal cycling comprised initial denaturation at 95°C for 2 min, followed by 25 cycles of denaturation at 94°C for 5 s, annealing at 55°C for 30 s, and elongation at 72°C for 30 s. PCR products from each sample were detected by electrophoresis on a 1.5% agarose gel. The obtained PCR products were purified, quantified, and mixed in equal amounts to construct sequencing libraries. Library quality was evaluated using an Agilent Bioanalyzer 2100 system and a Qubit @2.0 Fluorometer (Thermo Scientific). Finally, the Illumina NovaSeq 6000 platform (San Diego, CA, United States) was used to sequence the libraries using the 250 bp paired-end strategy.

### Data processing and bioinformatics analysis

Quality raw reads were filtered using QIIME v1.9.2 (Caporaso et al., 2010) to generate high-quality clean tags. Reads with an average Phred score (Q score) < 20 and those containing ambiguous bases, homopolymers >6, primer mismatches in, and sequence length <150 bp were deleted from the datasets (Bokulich et al., 2013). The remaining high-quality reads were assigned to the samples based on their unique barcodes at the ends of the reverse primers. Subsequently, reads with an overlap longer than 10 bp and without any mismatch were assembled into tags using FLASH (Magoč and Salzberg, 2011). These tags were then assigned to samples based on their unique barcodes and truncated by removing the barcodes and primer sequences. Chimeras were also distinguished and eliminated using the QIIME v. 1.9.2. Tags with ≥97% similarity were assigned to the same operational taxonomic unit (OTU) by UCLUST, using the open reference strategy (Edgar, 2010). The representative sequence of each OTU was chosen using the default method, and a bacterial taxon was assigned based on the SILVA database (Release 138; Yilmaz et al., 2013). A bacterial OTU abundance table was constructed and normalized using the standard number of tags according to the sample with the least number of tags (27,193 tags). Singletons (abundance <0.001%) were removed to improve the efficiency of data analysis (Bokulich et al., 2013). All subsequent analyses were performed using normalized OTU abundance data.

### Statistical analysis

Differences in sediment characteristics and fatty acids between the PG and CG groups were determined by conducting independent sample t-tests with a significance level of *p* = 0.05. Before statistical analysis, the normality of distributions and homogeneity of variance in the raw data were assessed using the Kolmogorov–Smirnov and Levene’s tests (Zar, 2007). Data are presented as the mean ± SD (*n* = 3). SPSS 22.0 and PRISM 8.0 were used for analysis and graphing, respectively. Differences in relative abundances of dominant bacterial phyla and genera and functional terms between the PG and CG groups were confirmed by conducting White’s non-parametric *t*-test using statistical analyses of metagenomic profiles (STAMP; Parks and Beiko, 2010). The Bray-Curtis distances among different samples were calculated. Principal component analysis (PCA), unweighted pair-group method with arithmetic means (UPGMA) hierarchical cluster analysis, and PERMANOVA tests based on Bray-Curtis distance were performed to assess the effects of sampling time and *P. clarkii* bioturbation on benthic bacterial community composition. Linear discriminant analysis (LDA) effect size (LEfSe) analysis was used to determine the statistical and biological differences in benthic bacterial communities between the PG and CG groups. Ecologically relevant functions of bacterioplankton communities were established using the functional annotation of prokaryotic taxa (FAPROTAX; Louca et al., 2016). Spearman’s correlation coefficients were assessed to determine the relationships between the bacterial communities, OM degradation states, and environmental factors. A correlation was considered significant when the absolute value of Spearman’s rank correlation coefficient (Spearman’s r) was >0.6 and statistically significant (*p* < 0.05).

## Results

### Sediment characteristics

**Figure 1:**
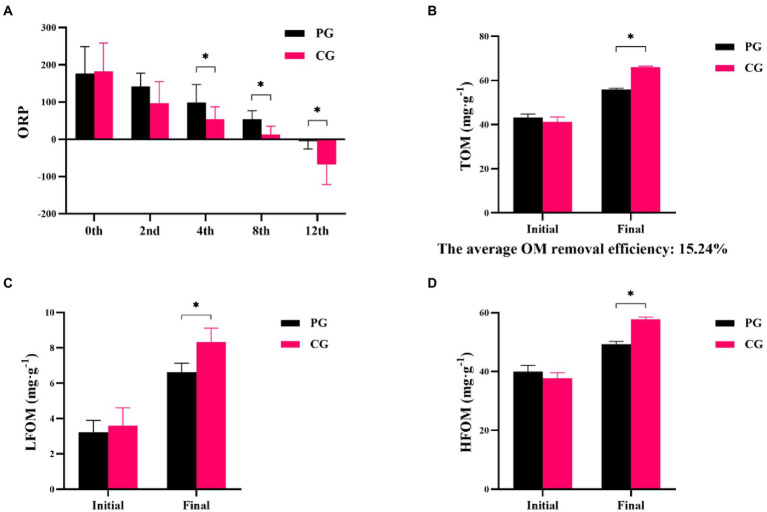
Differences in sediment characteristics between PG and CG groups during the experimental period, including oxidation–reduction potential (ORP; **A**), total organic matter (TOM; **B**), light fraction organic matter (LFOM; **C**), and heavy fraction organic matter (HFOM; **D**). “Initial” and “Final” indicate the initial and final stages of the experimental period, respectively. 0th, 2nd, 4th, 8th, and 12th indicate the initial stage, 2nd, 4th, 8th, and 12th week, respectively. * indicates a significant difference between PG and CG groups (*p* < 0.05).

Differences in ORP, TOM, LFOM, and HFOM between the PG and CG groups during the experimental period are shown in Figure 1. At the beginning of the experiment, no significant differences were observed in the ORP, TOM, LFOM, and HFOM between the PG and CG (*p* > 0.05). As the experiment progressed, significantly higher ORP values were found in the PG than in the CG at the 4th, 8th, and 12th weeks (*p* < 0.05). The TOM, LFOM, and HFOM contents in the PG were significantly lower than those in the CG at the end of the experiment (*p* < 0.05). The average organic matter removal efficiency in the PG group was 15.24%.

### Fatty acids and OM degradation state

**Figure 2:**
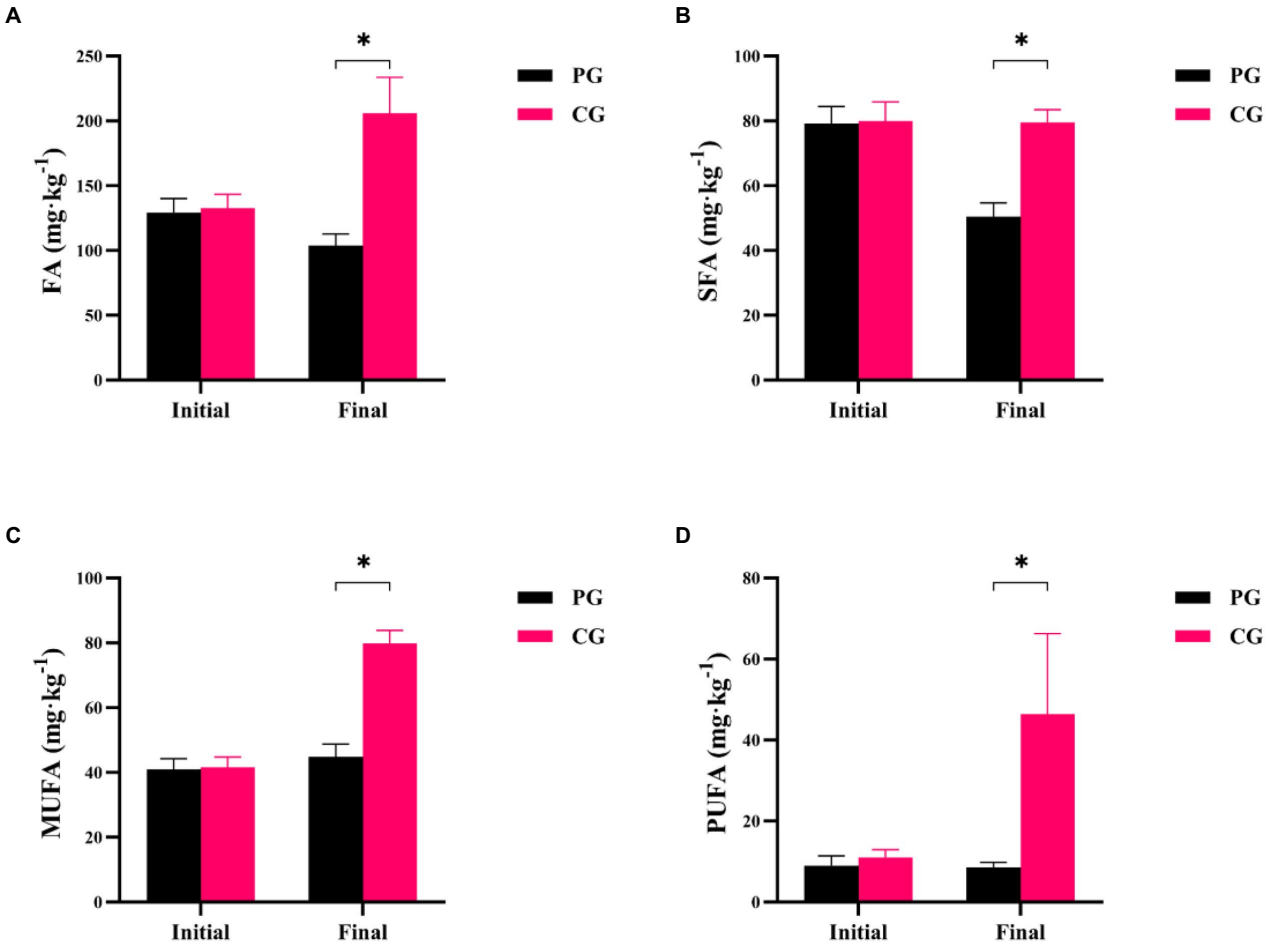
Differences in sedimentary fatty acids between PG and CG groups during the experimental period, including FA **(A)**, SFA **(B)**, MUFA **(C)**, and PUFA **(D)**. “Initial” and “Final” indicate the initial and final stages of the experimental period, respectively. * indicates a significant difference between PG and CG groups (*p* < 0.05).

Differences in FA, saturated fatty acids (SFA), monounsaturated fatty acids (MUFA), and polyunsaturated fatty acids (PUFA) between the PG and CG groups during the experimental period are shown in Figure 2. At the beginning of the experiment, there were no significant differences in FA, SFA, MUFA, and PUFA concentrations between the PG and CG (*p* > 0.05). However, at the end of the experiment, the PG showed significantly lower FA, SFA, MUFA, and PUFA contents in the sediment than the CG (*p* < 0.05).

**Figure 3:**
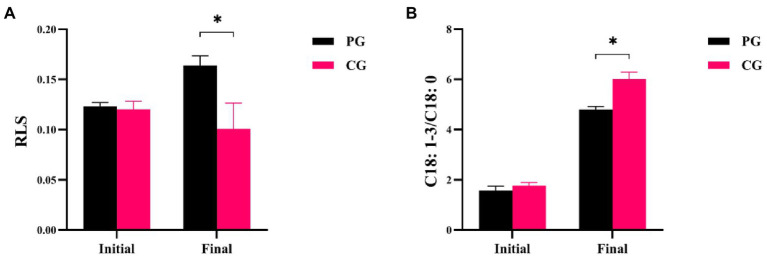
Differences in RLS **(A)** and C18: 1-3/C18: 0 **(B)** between PG and CG groups during the experimental period. “Initial” and “Final” indicate the initial and final stages of the experimental period, respectively. * indicates a significant difference between PG and CG groups (*p* < 0.05).

Differences in RLS and C18:1-3/C18:0 between the PG and CG groups during the experimental period are shown in Figure 3. The RLS and C18:1-3/C18:0 values of PG were not significantly different from those of CG at the initial time point, respectively (*p* > 0.05). However, when the experiment was completed, the RLS value of PG was significantly higher than that of CG, but the C18:1-3/C18:0 of PG was significantly lower (*p* < 0.05).

### Bacterial community structure

**Figure 4:**
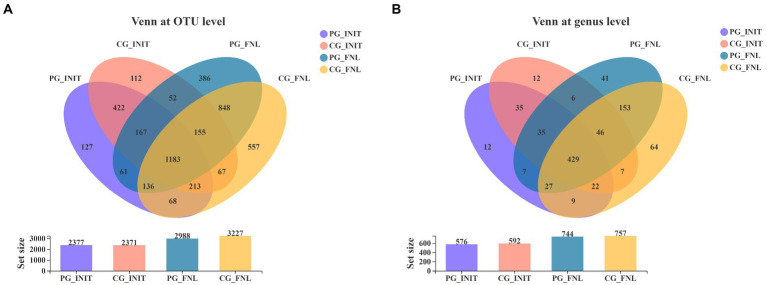
Venn diagrams of PG and CG groups at the initial and final stages of the experiment, including the Venn diagrams at the OTU level **(A)** and genus level **(B)**. PG_INIT and CG_INIT represented the PG and CG groups at the initial stage of the experimental period, respectively. PG_FNL and CG_FNL represented the PG and CG groups at the final stage of the experimental period, respectively.

Our study obtained 4,554 distinct OTUs from the sediment samples using Illumina sequencing technology based on the bacterial 16S rRNA gene, assigned to 43 phyla and 905 genera. As shown in Figure 4A, there were 392 OTUs unique to the PG group, 386 OTUs unique to the CG group, and 1985 OTUs shared by the PG and CG groups at the beginning of the experiment. When the experiment ended, the PG and CG groups contained 666 and 905 unique OTUs, respectively, along with 2,322 mutual OTUs. For the Venn diagram at the genus level (Figure 4B), the samples contained 55 genera unique to the PG group, 71 genera unique to the CG group, and 521 genera common to the PG and CG groups at the beginning. At the end of the experiment, 89 genera unique to PG, 102 genera unique to CG, and 655 genera common to PG and CG were observed in the samples.

**Figure 5:**
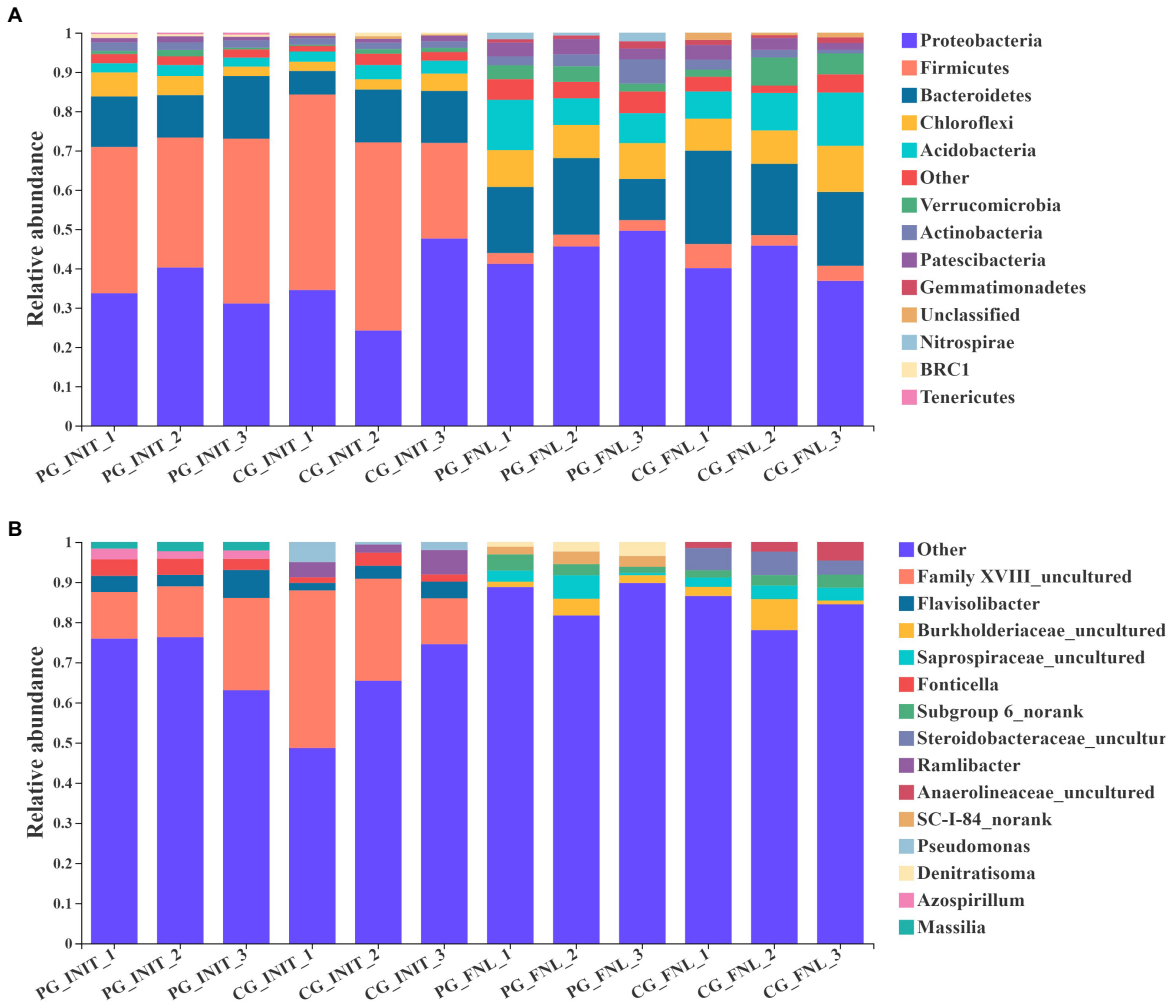
Relative abundances of the dominant phyla (**A**, most abundant top 10) and genera (**B**, most abundant top 5) in PG and CG groups at the initial and final stages of the experiment. PG_INIT and CG_INIT represented the PG and CG groups at the initial stage of the experimental period, respectively. PG_FNL and CG_FNL represented the PG and CG groups at the final stage of the experimental period, respectively.

The dominant phyla (most abundant top 10) and genera (most abundant top 5) in the sediment of the PG and CG groups during the experimental period are shown in Figure 5. Initially, nine of the most abundant top 10 phyla in the PG and CG groups were Firmicutes, Proteobacteria, Bacteroidetes, Chloroflexi, Acidobacteria, Actinobacteria, Patescibacteria, Verrucomicrobia, and BRC1. The remaining unique phyla were Tenericutes in the PG and unclassified in the CG. No significant differences in the relative abundances of Firmicutes, Proteobacteria, Bacteroidetes, Chloroflexi, Acidobacteria, Actinobacteria, Patescibacteria, Verrucomicrobia, or BRC1 were found between the PG and CG groups at the beginning of the experiment (*p* > 0.05). At the end of the experiment, there were also nine similar phyla of the most abundant top 10 phyla in the PG and CG groups, namely Proteobacteria, Bacteroidetes, Acidobacteria, Chloroflexi, Actinobacteria, Patescibacteria, Verrucomicrobia, Firmicutes, and Gemmatimonadetes. The relative abundances of the phyla Proteobacteria, Bacteroidetes, Acidobacteria, Chloroflexi, Actinobacteria, Patescibacteria, Verrucomicrobia, Firmicutes, and Gemmatimonadetes were similar between the PG and CG groups (*p* > 0.05). The only unique phylum of the most abundant top 10 phyla in the PG group was Nitrospirae, whereas the only unique phylum in the CG group was unclassified.

As for the dominant genera, three of the most abundant top 5 genera in the PG and CG groups were the same at the beginning of the experiment, namely *Family XVIII uncultured*, *Flavisolibacter*, and *Fonticella.* There were no significant differences in the relative abundances of *Family XVIII uncultured*, *Flavisolibacter*, or *Fonticella* between the PG and CG groups (*p* > 0.05). The genera *Massilia* and *Azospirillum* were unique to the PG group, whereas the genera *Ramlibacter* and *Pseudomonas* were unique to the CG group at the beginning of the experiment. When the experiment ended, three of the most abundant top 5 genera in the PG and CG groups were the same, namely *Burkholderiaceae uncultured*, *Steroidobacteraceae uncultured*, and *Anaerolineaceae uncultured*. The relative abundances of *Burkholderiaceae uncultured*, *Steroidobacteraceae uncultured*, and *Anaerolineaceae uncultured* in the PG group were similar to those in the CG group (*p* > 0.05). In addition, the genera *Subgroup 6 norank* and *SC-I-84 norank* were unique to the PG group, whereas the genera *Azospirillum* and *Saprospiraceae uncultured* were unique to the CG group.

**Figure 6:**
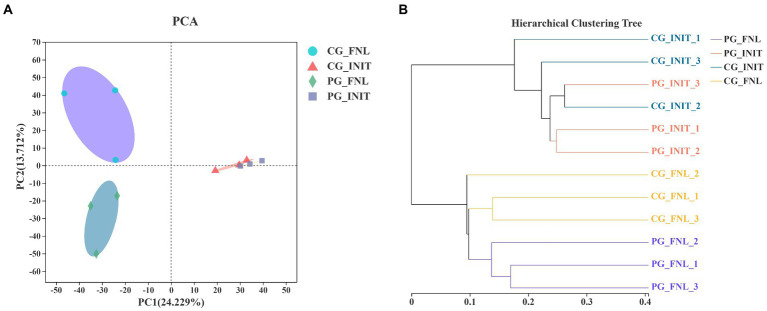
Principal component analysis (PCA; **A**) and unweighted pair-group method with arithmetic means (UPGMA) hierarchical cluster analysis **(B)** of the bacterial communities based on Bray-Curtis distance over all samples. PG_INIT and CG_INIT represent the PG and CG groups at the initial stage of the experimental period, respectively. PG_FNL and CG_FNL represent the PG and CG groups at the final stage of the experimental period, respectively.

Principal component analysis was applied to investigate the differences in sedimentary bacterial communities between the PG and CG groups based on the Bray-Curtis distance (Figure 6A). The first two PCs explained 24.23 and 13.71% of the total variation in the sedimentary bacterial communities. The bacterial communities of all sediment samples were categorized into three groups, as revealed by PCA results. Visible differences in the bacterial communities between the PG and CG groups were found at the end of the experiment. Two-way PEMANOVA also revealed that *P. clarkii* bioturbation and interaction between sampling time and *P. clarkii* bioturbation significantly impacted the bacterial communities in sediment (*p* < 0.05, Supplementary Table S2). A UPGMA hierarchical cluster analysis was also performed to confirm these results (Figure 6B). The UPGMA hierarchical cluster analysis initially divided all the samples into two categories, where the initial samples belonged to one category, and the final samples belonged to the other category. In the final sample category, PG_FNL_1, PG_FNL_2, and PG_FNL_3 were divided into separate categories that were differed from the CG_FNL_1, CG_FNL_2, and CG_FNL_3.

### Different bacterial taxa

**Figure 7:**
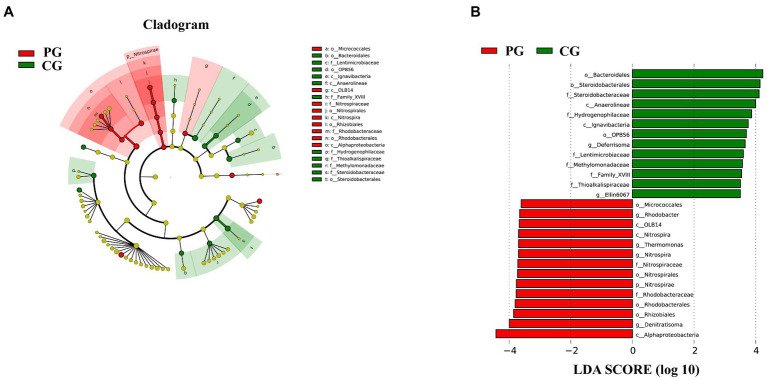
Linear discriminant analysis (LDA) effect size (LEfSe) analysis highlights the statistical and biological differences in sediment bacteria between PG and CG groups at the end of the experiment (LDA > 3.5), including the cladogram of the bacterial community **(A)** and the histogram of LDA scores **(B)**. Rings from the innermost to the outermost in the cladogram stand for the domain, phylum, class, order, family, and genus, respectively. The diameter of each circle in the cladogram reflects the abundance of that taxon in the community. Significant (red and green) and non-significant (yellow) discriminant taxonomic nodes are colored.

Linear discriminant analysis (LDA) effect size (LEfSe) analysis was used to clarify the statistical and biological differences in the sediment bacterial communities at each taxonomic level. LEfSe analysis determined 27 differential taxa with LDA values higher than 3.5, as shown in Figure 7. Fourteen differential taxa were enriched in the sediments of the PG. Notably, five differential taxa, namely Nitrospirae, Nitrospira, Nitrospirales, Nitrospiraceae, and *Nitrospira*, were organized from phylum to genus level, and four differential taxa, namely Alphaproteobacteria, Rhodobacterales, Rhodobacteraceae, and *Rhodobacter*, were organized from class to genus level. The other differential taxa were OLB14 (class level), Micrococcales (order level), Rhizobiales (order level), *Thermomonas* (genus level), and *Denitratisoma* (genus level). The CG group had 13 differential taxa enriched in the sediments: Anaerolineae (class level), Ignavibacteria (class level), Bacteroidales (order level), OPB56 (order level), Steroidobacterales (order level), Lentimicrobiaceae (family level), Methylomonadaceae (family level), Thioalkalispiraceae (family level), Family XVIII (family level), Hydrogenophilaceae (family level), Steroidobacteraceae (family level), *Deferrisoma* (genus level), and *Ellin6067* (genus level). As shown in Figure 7A, the differential taxa in the CG group were scattered and irregular, and no differential taxa at the continuous taxonomic level (e.g., from the phylum to genus level) were observed, similar to the PG group.

### Bacterial functional prediction

**Figure 8:**
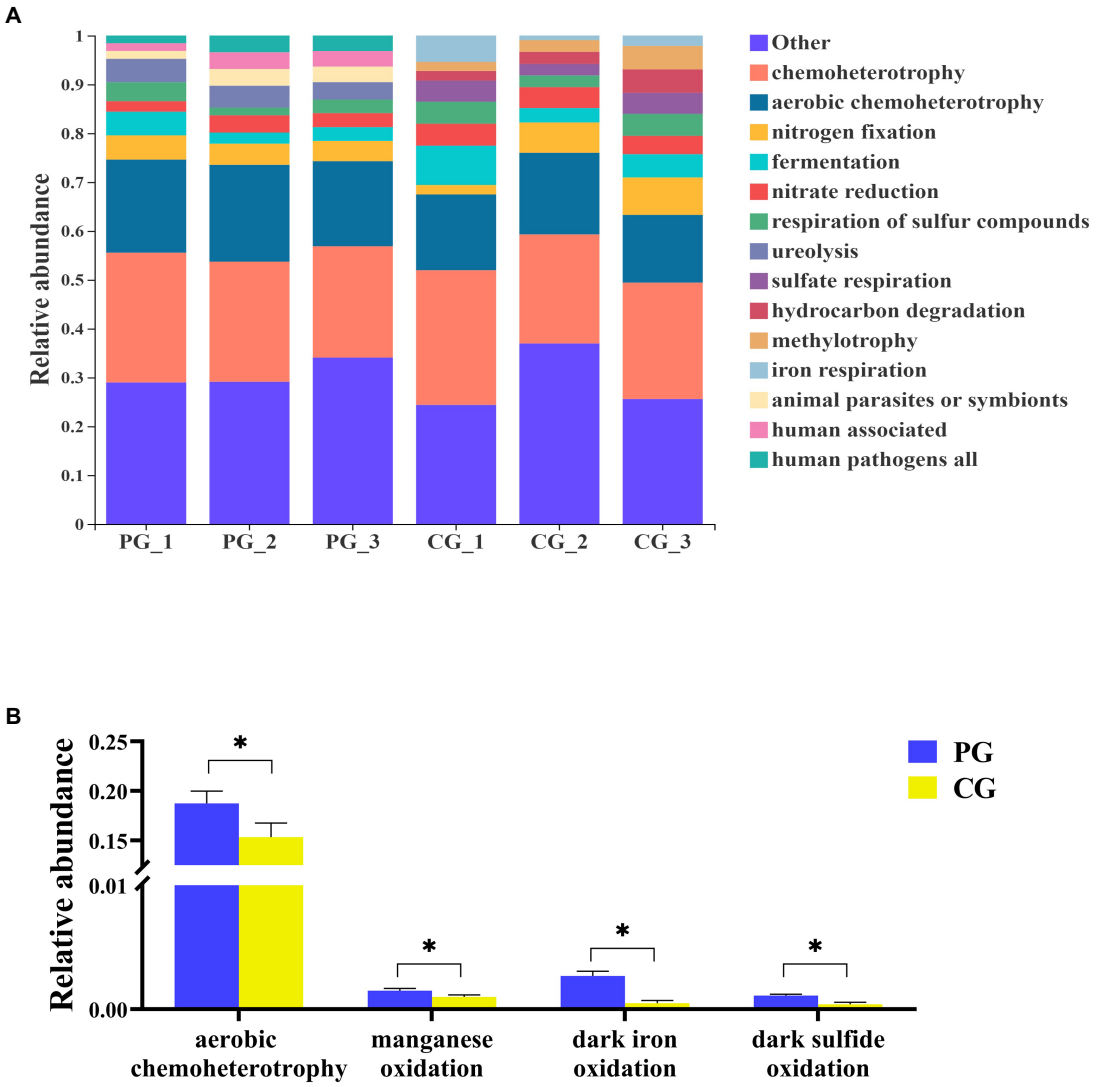
Relative abundances of the dominant functional groups (**A**, most abundant top 10) and the significantly different functional groups related to organic matter degradation **(B)** in PG and CG groups at the end of the experiment. * Indicates a significant difference between PG and CG groups (*p* < 0.05).

According to FAPROTAX, 69 functional groups in the PG and CG groups were predicted at the end of the experiment. The relative abundances of the dominant functional groups (most abundant top 10) and the significantly different functional groups related to OM degradation between the PG and CG are shown in Figure 8. At the end of the experiment, the dominant functional groups of the PG group were chemoheterotrophy (24.65 ± 1.54%), aerobic chemoheterotrophy (18.76 ± 1.01%), nitrogen fixation (4.48 ± 0.35%), ureolysis (4.28 ± 0.52%), fermentation (3.31 ± 1.10%), nitrate reduction (2.85 ± 0.56%), respiration of sulfur compounds (2.75 ± 0.95%), animal parasites or symbionts (2.74 ± 0.81%), human associated (2.73 ± 0.81%), and human pathogens all (2.72 ± 0.82%). And the dominant functional groups of CG group were chemoheterotrophy (24.59 ± 2.21%), aerobic chemoheterotrophy (15.35 ± 1.17%), nitrogen fixation (5.27 ± 2.43%), fermentation (5.25 ± 2.12%), nitrate reduction (4.18 ± 0.31%), respiration of sulfur compounds (3.81 ± 0.99%), sulfate respiration (3.66 ± 0.94%), hydrocarbon degradation (3.08 ± 1.22%), methylotrophy (3.02 ± 1.28%), and iron respiration (2.82 ± 1.89%).

To further determine the differences in the functional groups related to OM degradation between the PG and CG groups at the end of the experiment, an White’s non-parametric *t*-test was employed. As a result, four significantly different functional groups related to OM degradation were identified. The relative abundances of aerobic chemoheterotrophy, manganese oxidation, dark iron oxidation, and dark sulfide oxidation in the PG group were significantly higher than those in the CG group (*p* < 0.05).

### Correlations analysis

**Figure 9:**
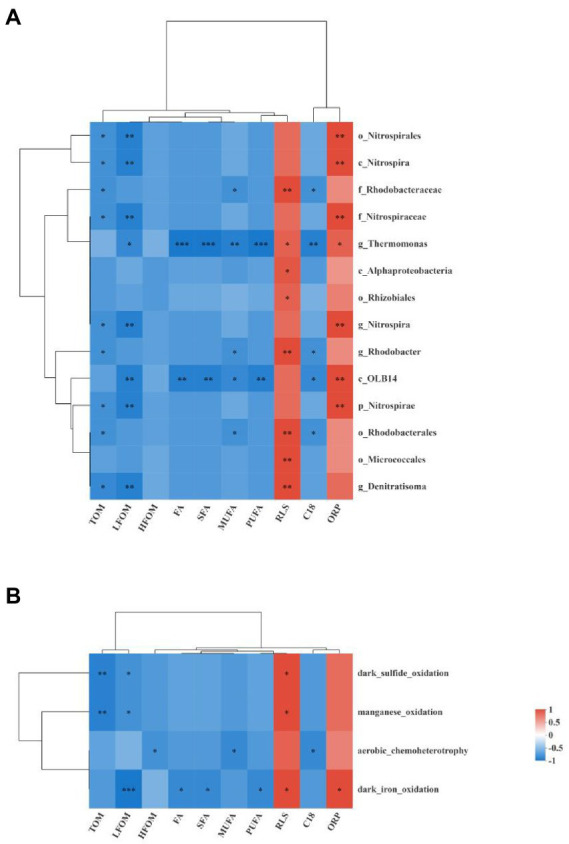
Heat maps show the Spearman correlation analysis between the different bacterial taxa enrich in the PG group and the environmental factors **(A)** and the Spearman correlation analysis between the different functional group and the environmental factors **(B)**. *, **, and *** denote significance at 0.05, 0.01, and 0.001 level, respectively. C18 indicated the C18:1-3/C18:0 ratio.

Spearman correlation analyses were used to determine the correlations between different bacterial taxa and environmental factors, as shown in Figure 9A. All 14 different bacterial taxa determined by LEfSe analysis were negatively correlated with TOM, LFOM, HFOM, FA, SFA, MUFA, PUFA, and C18:1-3/C18:0 ratio and were positively correlated with RLS and ORP. Among them, more than half of the differential bacterial taxa were significantly negatively correlated with TOM and LFOM and were significantly positively correlated with RLS and ORP (*p* < 0.05). The genus *Thermomonas* and class OLB14 were significantly correlated with almost all the environmental factors (*p* < 0.05). The order Rhodobacterales, family Rhodobacteraceae, and genus *Rhodobacter* were significantly negatively correlated with MUFA and C18:1-3/C18:0 ratio and were significantly positively correlated with RLS (*p* < 0.05).

The correlations between different functional groups and environmental factors are shown in Figure 9B. All the different functional groups were negatively correlated with TOM, LFOM, HFOM, FA, SFA, MUFA, PUFA, and C18:1-3/C18:0 ratio and were positively correlated with RLS and ORP. Among them, manganese oxidation and dark sulfide oxidation were significantly negatively correlated with TOM and LFOM and were significantly positively correlated with RLS (*p* < 0.05). Aerobic chemoheterotrophy was significantly negatively correlated with HFOM, MUFA, and C18:1-3/C18:0 ratio (*p* < 0.05). Dark iron oxidation was significantly negatively correlated with LFOM, FA, SFA, and PUFA and was significantly positively correlated with RLS and ORP (*p* < 0.05).

## Discussion

### Sediment characteristics and OM degradation state

Fatty acids, as one of the most abundant lipid biomarkers in sediments, are widely applied in studies focusing on the source and preservation of OM and on evaluating the “freshness” of OM in marine and freshwater environments (Dalsgaard et al., 2003; Yunker et al., 2005; Niggemann and Schubert, 2006; Li F. et al., 2018, Li et al., 2020; Feng et al., 2021). In particular, short-chain saturated and unsaturated fatty acids are easily degradable OM, the contents of which can indicate the degradation state of OM in sediments to a certain extent (Belicka et al., 2002; Yunker et al., 2005; Li et al., 2020). In the present study, significantly decreased FA, SFA, MUFA, and PUFA contents in the sediment of the PG group revealed more extensive degradation of OM under red swamp crayfish bioturbation. In addition, the RLS and C18:1-3/C18:0 ratio based on FA composition also characterize the overall degradation state of OM, where higher RLS values and low C18:1-3/C18:0 ratios reflect the preferential degradation of short-chain saturated and unsaturated fatty acids, respectively (Li F. et al., 2018). Hence, the results of RLS, C18:1-3/C18:0 ratio, FA, SFA, MUFA, and PUFA in the present study were consistent with each other and jointly revealed the significant promotion of sedimentary OM degradation by red swamp crayfish. Although the OM contents in the sediments of the PG and CG groups both increased with time, the OM content of the PG group was significantly reduced compared to that of the CG group. The significantly decreased TOM, LFOM, and HFOM contents in the sediment of the PG group could be explained by the extensive OM degradation caused by the red swamp crayfish.

Previous study reported that oxygen is one of the key factors affecting OM degradation. The participation of oxygen, even brief exposure to O_2_, is very important for promoting net remineralization and reoxidation of OM (Aller, 1994). However, macrobenthos can influence local environmental factors (e.g., O_2_) at the sediment–water interface, the bioturbation of which can increase the contact area and promote material transportation between sediment and upper water bodies, thus effectively transporting oxygen into the sediment and increasing supply of electron acceptors (Massin, 1982a,b; De Bund et al., 1994; Aller, 2001; Grenz et al., 2003; Sanz-Lázaro and Marín, 2011; Foshtomi et al., 2015). Increased availability of terminal electron acceptors in sediment can result in variations in oxidation–reduction potential, heralding more efficient OM degradation in the sediment (Angeler et al., 2001). Hence, the significantly increased ORP values of the PG group in the present study implied a significant promotion of red swamp crayfish on the oxygen transportation from overlying water into the sediment, successfully creating a more favorable condition for OM degradation in sediment. In addition, the red swamp crayfish is a typical omnivorous benthic organism, the main food resources of which are organic debris, fresh macrophytes, and sediment grains (Geiger et al., 2004). Accordingly, the significantly higher degree of OM degradation and lower OM content in the sediment of the PG group could be attributed to the enhanced oxygen supply and direct consumption associated with crayfish activities. However, OM degradation process and respiration of red swamp crayfish all consume oxygen. The balance between oxygen input and consumption has an important influence on the OM degradation and removal. The promotion of red swamp crayfish on the OM degradation confirmed in this experiment may be seriously affected and limited in actual culturing production, especially in the case of insufficient oxygen supply.

### Bacterial community structure and functional composition

Organic matter degradation in sediments is essentially a bacterial process in which heterotrophic bacteria play a key role (Freixa et al., 2016). Previous studies have reported that benthic fauna bioturbation can affect the metabolism, activity, diversity, and composition of bacterial communities in sediments (Mermillodblondin et al., 2004; Laverock et al., 2010; Rugenski et al., 2012; Hou et al., 2021a). In the present study, differences in PCA, hierarchical cluster analysis, and two-way PEMANOVA analysis revealed significant impacts of red swamp crayfish on benthic bacterial communities. Furthermore, the numerical and biological differences in sediment microflora between PG and CG groups determined by LEfSe analysis also confirmed that red swamp crayfish affected the benthic bacterial communities. Redox conditions and availability of OM and nutrients can be the main driving factors causing considerable variations in bacterial community composition and function, especially at small environmental scales (Jensen et al., 1993; Urakawa et al., 2000; Köster et al., 2005, 2008; Böer et al., 2009; Jansen et al., 2009; Laverock et al., 2010). The enhanced oxygen transportation and increased ORP values resulting from red swamp crayfish bioturbation in the sediments of the PG group have been discussed above. The enhanced oxygen transportation and increased ORP values can extend the oxic-anoxic interface, affect microbial redox reactions, and increases heterotrophic activity of both aerobic and anaerobic bacteria (Papaspyrou et al., 2005; Laverock et al., 2010). In addition, benthos can improve microbial OM availability by converting large organic particles into smaller ones which are more easily utilized by microorganisms (Levin et al., 1997; Kristensen and Holmer, 2001; Kristensen et al., 2012). As described by Dadi et al. (2017) and Palmas et al. (2019), red swamp crayfish bioturbation can contribute to sediment resuspension, thereby increasing the microbial potential to utilize organic substrates. Accordingly, enhanced ORP conditions and increased microbial OM availability could be the main mechanism by which red swamp crayfish affected the microbial community structure in the sediment. The significant positive correlations between the ORP and most bacterial taxa significantly enriched in the PG group also supported this conclusion.

In the present study, Nitrospirae Nitrospira Nitrospirales Nitrospiraceae *Nitrospira*, Alphaproteobacteria Rhodobacterales Rhodobacteraceae *Rhodobacter*, OLB14, Micrococcales, Rhizobiales, *Thermomonas*, and *Denitratisoma* were significantly enriched in sediments of the PG group. *Nitrospira* plays an important role in nitrification (Daims et al., 2015). As described in previous studies, *Nitrospira* is not only a nitrite-oxidizing bacterium, but also seems to be able to achieve complete ammonia oxidation (Daims et al., 2011, 2015; Daebeler et al., 2014). *Rhodobacter* is a heterotrophic nitrification-aerobic denitrification bacterium that involved in OM decomposition and nitrogen removal (Yang et al., 2019; Xiang et al., 2020). *Thermomonas* and *Denitratisoma* are aerobic denitrifying bacteria that contribute to OM degradation and nitrogen removal (McIlroy et al., 2016; Wu et al., 2016; Chen et al., 2018; Xia et al., 2019; Tao et al., 2022). Micrococcales and Rhizobiales also participate in OM degradation (Liu et al., 2020; Zhang et al., 2021; Zou et al., 2022). In addition, most bacterial taxa that were significantly enriched in the PG group showed significant correlations with OM degradation indicators, especially negative correlations with TOM and LFOM, and a positive correlation with RLS. These results also imply a significant contribution to OM degradation from the significantly enriched bacterial taxa in the PG group. Hence, the bacterial communities in the sediment of the PG group, influenced by red swamp crayfish, demonstrated stronger abilities to degrade and utilize organic matter and remove nitrogen.

Results related to bacterial community functions in the sediment of the PG group also supported our conclusion. The bacterial community functions related to OM degradation, namely aerobic chemoheterotrophy, manganese oxidation, dark iron oxidation, and dark sulfide oxidation, were significantly enriched in the sediment of the PG group. Abundant aerobic chemoheterotrophy generally indicates a large number of bacteria that obtain energy through OM aerobic oxidation, and chemoheterotrophic bacteria usually act as OM decomposers and play a vital role in environmental remediation, OM recycling, and the carbon cycle in ecosystems (Kämpfer et al., 1993; Zhang et al., 2018). Meanwhile, the manganese oxidation function can provide nutrient sources for bacteria and facilitate the bacterial utilization of organic matter (Duan et al., 2020). Manganese-oxidizing bacteria obtain energy mainly by decomposing organic substrates for chemoheterotrophic growth (Zhao et al., 2015). Dark iron oxidation can form hydroxyl radicals through the oxidation of Fe^2+^ *via* Fenton-type reactions, which play a critical role in OM degradation and transformation (Zhu et al., 2019; Lomardo et al., 2020). Dark sulfide oxidation can also form hydroxyl radicals *via* Fenton-like chemistry, thus contributing to OM degradation (Lomardo et al., 2020). The important roles of aerobic chemoheterotrophy, manganese oxidation, dark iron oxidation, and dark sulfide oxidation in OM degradation could also be confirmed by their significant relationship with OM degradation indicators. Accordingly, red swamp crayfish could significantly promote the bacterial abilities for OM decomposition in sediment by altering the bacterial composition and functional groups.

## Conclusion

In summary, after the 84-day indoor simulation experiment, the red swamp crayfish *P. clarkia* effectively increased the ORP values of sediments to provide favorable conditions for OM degradation and changed the composition and function of bacterial communities to improve bacterial abilities for OM decomposition and utilization, thereby promoting OM degradation in sediment. Furthermore, by enhancing OM degradation, the red swamp crayfish significantly reduced TOM, LFOM, and HFOM concentrations and successfully inhibited OM accumulation in the sediment. From these aspects, red swamp crayfish have the potential to alleviate excessive OM accumulation in sediments and reduce the risk of endogenous water pollution and eutrophication in aquaculture ponds.

Since field culture experiments are always influenced by many difficult environmental factors to control, we chose to conduct an indoor simulation experiment in this study. However, the indoor simulation experiment was more ideal than an actual pond culture environment. Furthermore, limited by the small water area and low anti-interference performance of indoor simulation experiments, we did not conduct continuous-time gradient sampling in this experiment to maintain the strict single-factor influence and avoid the interference of artificial sampling operations on OM degradation. Although this ensured the rigor of the experiment, it also resulted in a lack of continuous observation and understanding of the OM degradation processes in sediments. Hence, we will combine indoor simulation and field culture experiments in future to further confirm and enrich our findings.

## Data availability statement

The datasets presented in this study can be found in online repositories. The names of the repository/repositories and accession number(s) can be found at: https://www.ncbi.nlm.nih.gov/, PRJNA849328.

## Author contributions

YH: conceptualization, methodology, formal analysis, data curation, and writing—original draft preparation. RJ and PJ: visualization and investigation. BL: visualization and writing reviewing and editing. JZ: resources, writing—reviewing and editing, and supervision. All authors contributed to the article and approved the submitted version.

## Funding

This work was financially supported by the Central Public-interest Scientific Institution Basal Research Fund, CAFS (grant no. 2022XT0504), the China Agriculture Research System of MOF and MARA (grant no. CARS-45), and the National Natural Science Foundation of China (grant no. 31802302).

## Conflict of interest

The authors declare that the research was conducted in the absence of any commercial or financial relationships that could be construed as a potential conflict of interest.

## Publisher’s note

All claims expressed in this article are solely those of the authors and do not necessarily represent those of their affiliated organizations, or those of the publisher, the editors and the reviewers. Any product that may be evaluated in this article, or claim that may be made by its manufacturer, is not guaranteed or endorsed by the publisher.

## Supplementary material

The Supplementary material for this article can be found online at: https://www.frontiersin.org/articles/10.3389/fmicb.2022.985555/full#supplementary-material
