# Supplementary material for: Organic matter degradation and bacterial communities in surface sediment influenced by Procambarus clarkia
Source: Front Microbiol. 2022 Oct 21;13:985555. doi: 10.3389/fmicb.2022.985555 (PMC9634481; doi:10.3389/fmicb.2022.985555)
Supplement: Supplementary file 1 [file Data_Sheet_1.docx]

**Supplementary Material for**

**Organic matter degradation and bacterial communities in surface sediment influenced by *Procambarus clarkia***


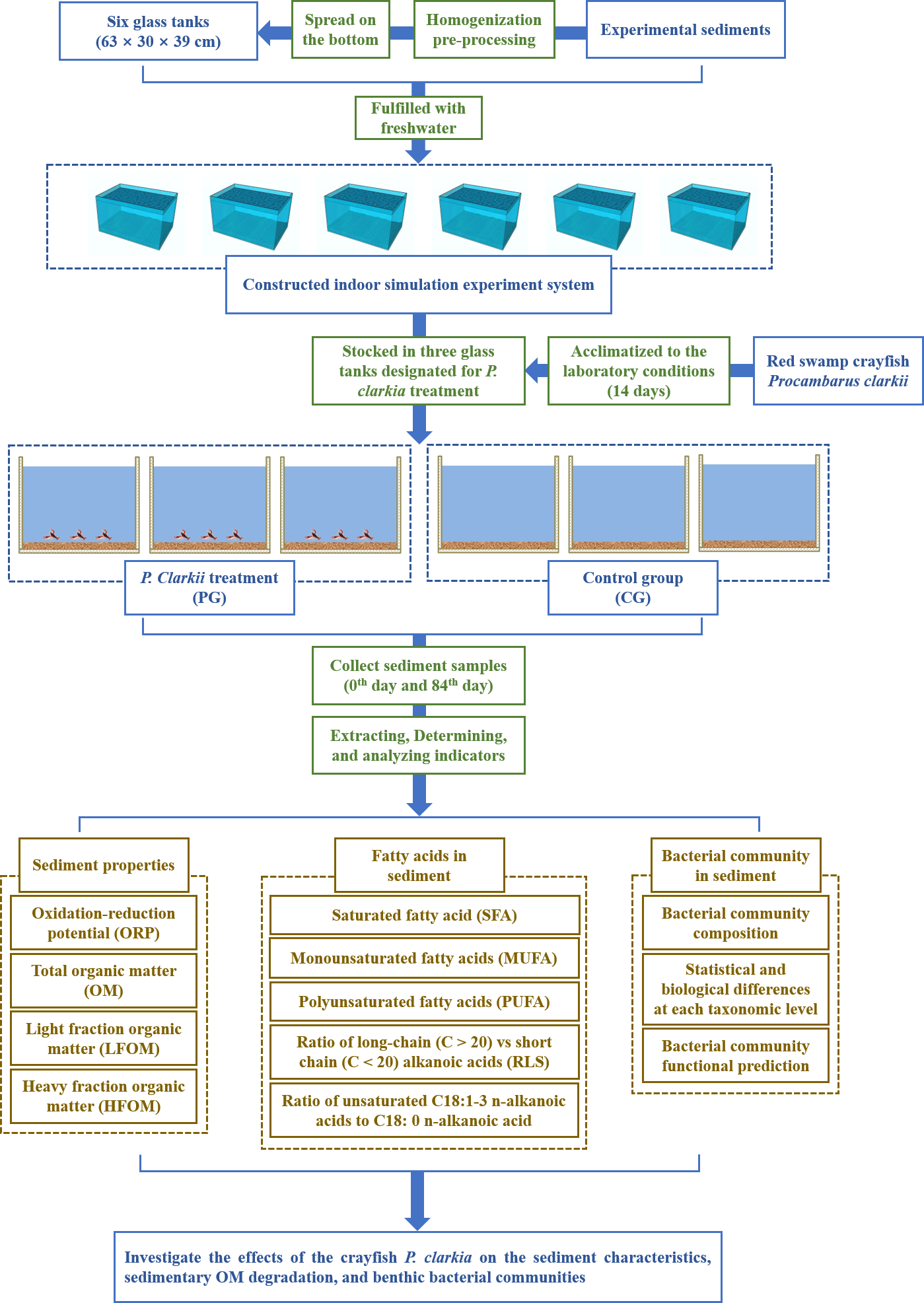


**Figure S1.** Experimental flowchart

**Table S2.** Two-way PERMANOVA of sampling time and *Procambarus clarkii* bioturbation for bacterial community based on the Bray-Curtis distance.

|  | **F** | **R^2^** | **p-value** |
| --- | --- | --- | --- |
| **Sampling time** | 0.7247 | 0.0676 | 0.582 |
| ***P. clarkii* bioturbation** | 10.7670 | 0.5185 | 0.002^**^ |
| **Sampling time: Mode** | 5.0262 | 0.6534 | 0.001^***^ |
